# Involvement of the MiR-181b-5p/HMGB1 Pathway in Ang II-induced Phenotypic Transformation of Smooth Muscle Cells in Hypertension

**DOI:** 10.14336/AD.2018.0510

**Published:** 2019-04-01

**Authors:** Feng-Juan Li, Cheng-Long Zhang, Xiu-Ju Luo, Jun Peng, Tian-Lun Yang

**Affiliations:** ^1^Department of Cardiovascular Medicine, Xiangya Hospital, Central South University, Changsha 410008, China.; ^2^Department of Laboratory Medicine, Xiangya School of Medicine, Central South University, Changsha410013, China.; ^3^Department of Pharmacology, Xiangya School of Pharmaceutical Sciences, Central South University, Changsha, China.; ^4^Hunan Provincial Key Laboratory of Cardiovascular Research, Xiangya School of Pharmaceutical Sciences, Central South University, Changsha 410078, China

**Keywords:** HMGB1, hypertension, phenotypic transformation, miR-181b-5p

## Abstract

Phenotypic transformation of vascular smooth muscle cells (VSMCs) contributes to vascular remodeling in hypertension. High mobility group box-1 (HMGB1) has been reported to be involved in several pathogenic processes including VSMC proliferation and migration. The present study was designed to determine the role of HMGB1 in VSMC phenotypic transformation in hypertension. First, we demonstrated that HMGB1 was elevated in a model of Ang II-induced VSMC phenotypic transformation, which showed down-regulation of contractile proteins and up-regulation of synthetic proteins. Knockdown of HMGB1 and losartan could block the phenotypic transformation. Next, we identified three potential miRNAs for upstream regulation of HMGB1 by bioinformatic analysis; only miR-181b-5p was significantly down-regulated in Ang II-treated cells. Co-treating the cells with miR-181b-5p mimics suppressed HMGB1 expression as well as the phenotypic transformation, migration, and proliferation. Furthermore, the luciferase reporter gene assay confirmed the direct interaction between miR-181b-5p and HMGB1. Finally, to extend these cell-based studies to clinical patients, we demonstrated that plasma miR-181b-5p levels were decreased, while Ang II and HMGB1 levels, as well as the intima-media thickness (IMT) were increased in hypertensive patients; these effects were reversed following the administration of angiotensin receptor blockers. Based on these observations, we conclude that the down-regulation of miR-181b-5p leads to the elevation of HMGB1 levels in hypertensive patients, which accounts, at least partially, for VSMCs phenotypic transformation and vascular remodeling. Our findings also highlight that the plasma levels of miR-181b-5p and HMGB1 may serve as novel biomarkers for vascular remodeling in the hypertensive patients.

Hypertension is a common disease and a wide-spread public-health challenge. Over a third of people worldwide have been diagnosed with hypertension [1]. In China, hypertension is considered as a major chronic non-infectious disease and the most important risk factor for cardiovascular diseases. The prevalence of hypertension is >30% and the costs represent approximately 6.61% of the total healthcare budget [2-4]. Extensive efforts have been made to improve the treatment rate for hypertension; however, only approximately 20% of patients received the treatment [4]. Thus, more studies examining the pathogenesis, effective diagnosis, and low-cost treatments of hypertension remain necessary.

Several mechanisms are responsible for the development of hypertension including excessive vasoconstriction and/or deficient vasodilatation [5]. The remodeling of large and small arteries contributes to the development and complications of hypertension. It is well known that the renin-angiotensin system (RAS) plays a critical role in the development of hypertension and end-organ damage. Angiotensin II (Ang II) is known as a potent vasoconstrictor regulator that elevates blood pressure via activation of the mitogen activated protein kinase (MAPK) signaling pathway. Therefore, angiotensin-converting enzyme inhibitors and angiotensin receptor blockers are used to reduce blood pressure [6-8].

Vascular smooth muscle cells (VSMCs) are a dominant constituent of arteries and a critical determinant of vascular diseases [9]. VSMCs may undergo phenotype alternations between a differentiated phenotype (contractile phenotype) and a dedifferentiated phenotype (synthetic phenotype) in response to different stimuli. The contractile phenotype is characterized by high expression of contractile genes with a low rate of proliferation and migration. Conversely, synthetic VSMCs express low levels of contractile genes with a high rate of proliferation and migration [10, 11]. Thus, VSMCs phenotypic transformation from contraction to synthesis is widely accepted as the pivotal process in vascular remodeling during hypertension[12].However, the mechanisms responsible for VSMCs phenotypic transformation in hypertension are not fully understood.

Previous studies have demonstrated that low-grade inflammation is involved in the development of hypertension in humans and experimental models. Ang II has been shown to induce inflammatory reactions and Ang II-induced VSMCs phenotypic transformation contributes to the development of hypertension [13-17], suggesting a possible link between Ang II-induced VSMCs phenotypic transformation and inflammation. Recently, high mobility group box-1 (HMGB1) has been proposed as a potent pro-inflammatory cytokine that participates in the development of cardiovascular diseases [18-20]. For example, HMGB1 levels are elevated in atherosclerotic plaques, promoting VSMCs migration and proliferation [5, 21, 22]. Based on these reports, it is reasonable to speculate that HMGB1 plays a role in the mediation of Ang II- induced VSMCs phenotypic transformation in hypertension.

MicroRNAs (miRNAs), small non-coding RNAs, are well-known as negative regulators of the mRNA expression of target genes [6, 23]. Certain miRNAs have been shown to regulate VSMCs functions such as migration and proliferation [24, 25]. Abnormal expression of miRNAs is involved in a variety of human diseases including coronary heart diseases, hypertension, and vascular aneurysms [26, 27]. However, it is unknown whether certain miRNAs are involved in the Ang II-induced VSMCs phenotypic transformation in hypertension.

The goals of this study were as follows: 1) to investigate the roles of HMGB1 in Ang II-induced VSMCs phenotypic transformation, migration, and proliferation; 2) to identify miRNAs involved in the regulation of HMGB1 expression; and 3) to determine whether the miRNAs/HMGB1pathway is involved in vascular remodeling in hypertension patients with high plasma Ang II levels.

## MATERIALS AND METHODS

### In vitro study

#### Protocol for cell experiments

Human aortic vascular smooth muscle cells (HAVSMCs) were obtained from the Central South University Cell Center and cultured in high glucose Dulbecco's Modified Eagle's Medium (DMEM, Biological Industries, israel) containing 10% fetal bovine serum (FBS, Biological Industries, israel). Cells were incubated at 37 °C in a humidified atmosphere of 95% air and 5% CO_2_ and sub-cultured every 3 or 4 days with trypsin (0.25%) added EDTA (0.01%).

To explore the role of HMGB1 in Ang II-induced phenotypic transformation of HAVSMCs, HAVSMCs were randomly divided into three groups: control, without any treatment; Ang II, cells incubated with Ang II (1μM) for 48h to induce phenotypic transformation; and Ang II + losartan, cells pretreated with losartan (10 μM) for 1h before prior to Ang II treatment.

To confirm the function of HMGB1 in mediating the Ang II-induced phenotypic transformation of HAVSMCs, HAVSMCs were randomly divided into four groups: the control group, the Ang II group, the HMGB1 siRNA group (cells transfected with 50 nM of the HMGB1siRNA for 24h prior to Ang II treatment), and the siRNA negative control (NC) group (cells transfected with 50nM of the siRNA NC for 24h prior to Ang II treatment).

To determine whether miR-181b-5p is an upstream regulator of HMGB1 in Ang II-treated HAVSMCs, HAVSMCs were randomly divided into four groups: the control group, the Ang II group, the miR-181b-5p mimic group (cells transfected with 50nM of the miR-181b-5p mimic for 24h prior to Ang II treatment), and the mimic NC group (cells were transfected with 50 nM of the mimic NC for 24h prior to Ang II treatment).

At the end of the experiments, cells were collected for analysis of parameters relevant to phenotypic transformation and molecular studies (miRNA, mRNA, or protein expression).

**Table 1:** The primary antibodies for Western Blot.

Product Number	Protein Name	Dilution ratio	Manufacturers
ab79823	HMGB1	1:10000	abcam, USA
ab32575	α-SMA	1:1000	abcam, USA
ab14106	SM22α	1:1000	abcam, USA
ab91655	OPN	1:5000	abcam, USA
A531	GAPDH	1:10000	Bioworld, USA

#### Cell transfection experiments

The human HMGB1 small interference RNA (siRNA), miR-181b-5p mimic, NC siRNA, NC mimic, and transfection reagent were all purchased from RiboBio (Guangzhou, China). qPCR assays were used to determine the transfection efficiency. HAVSMCs grown to 50% to 60% confluence were starved using high glucose DMEM with 0.5% FBS for 24h and then transfected using the riboFECTTM transfection system for 24h. Prior to the above-mentioned experiments, all of the miRNA mimics and HMGB1 siRNAs used in this study were confirmed to work as expected. All transfections followed the manufacturer's instructions.

#### Cell migration assay

HAVSMCs migration was evaluated using wound-healing and Transwell migration assays. HAVSMCs were seeded in 6-well plates and the monolayers were scratched using sterile pipette tips. The cells were washed with PBS and then fresh DMEM with 0.5% FBS was added. The wound gaps were recorded using bright-field microscopy at 0h and 48h and measured with the Image-pro plus 6.0 software. The migration distance was analyzed by initial wound distance subtracting the remaining distance.

For the Transwell migration assays, HAVSMCs were seeded at a density of 1×10^5^ cells/well into the upper chambers, containing a filter membrane (8-μm pore size) of 24-well Transwell plates (Corning Inc., New York, USA). The lower chambers were then filled with 0.5% FBS medium containing 20 ng/ml platelet-derived growth factor-BB (PDGF-BB; PeproTech, USA) and the plate was further incubated for 12h. Migrated cells on the bottom of the filter were fixed with 4% triformol then stained using a crystal violet solution (Beyotime, Nanjing, China) and imaged by bright-field microscopy.

#### Cell proliferation assay

VSMCs proliferation was evaluated using the Edu incorporation assay according to the manufacturer’s instructions (RiboBio, R11053.2, Guangzhou, China). Briefly, HAVSMCs were seeded at a density of 6×10^3^ cells/well in 96-well plates and incubated with Edu for 2h, then fixed, stained, and imaged by fluorescence microscopy. The Edu-positive cells were counted and normalized to the total number of DAPI-stained cells [28].

#### Cell viability

Cell viability was evaluated using Cell Counting Kit-8 (CCK-8; Bimake, USA) according to the manufacturer's protocol. Briefly, HAVSMCs were seeded at a density 6×10^3^ cells/well in 96-well plates and incubated with the CCK-8 solution for 1h. The absorbance of the samples was measured at 450 nm using a microplate reader. Blank wells with 0.5% FBS were used at the blank control group.

#### Protein extraction and western blot analyses

Total proteins were extracted from HAVSMCs using RIPA lysis buffer (Beyotime, Nanjing, China). The BCA Protein Assay Kit (Beyotime, Nanjing, China) was used to determine the total protein concentrations. A standardized amount of total protein (20-40μg) from each sample was separated using 12% sodium dodecyl sulfate polyacrylamide gel electrophoresis. Following separation, proteins were transferred onto polyvinylidenefluoride membranes (Millipore, USA) and blocked with 5% non-fat milk at room temperature for 1h. The membranes were washed with TBST three times then incubated with primary antibodies at 4 °C overnight. After three washes, the membranes were incubated with peroxidase-conjugated affinipure goat anti-rabbit igg secondary antibody (ZB-5301, ZSGB-BIO, Beijing, China) at room temperature for 1h. The chemiluminescence signals were detected using the BeyoECL Star Kit (Beyotime, Nanjing, China). Densitometric analysis was conducted using Image J 1.43 software (National Institutes of Health New York City, NY, USA). GAPDH was used as the internal control. All primary antibody manufacturers and concentrations used in these experiments are detailed in Table 1.

#### RNA extraction and quantitative real-time polymerase chain reaction

Total RNA was extracted from the HAVSMCs using the Trizol reagent (Invitrogen, Waltham, MA, USA). RNAs enriched for small size were extracted from 200 μl of plasma using the EasyPure® miRNA Kit (ER601, TransGen Biotech, Beijing, China) according to the manufacturer's protocol; 5µl of 5 nM Syn-cel-miR-39 miScript miRNA Mimic (RiboBio) were added to each sample prior to the addition of acid-phenol:chloroform [29].

**Table 2:** Human Primer sequences for quantitative real-time PCR.

Gene	Forward (F) and reverse (R)
GAPDH	F:5'-TGATGACATCAAGAAGGTGGTGAAG R:3'-TCCTTGGAGGCCATGTGGGCCAT
α-SMA	F:5'- AGCGTGGCTACCCTTCGTGAC R:3'-GCTCGTTGCCGATGGTGATGAC
SM22α	F:5'-GAATGGCGTGATTCTGAGC R:3'-CTCCATCTGCTTGAAGAC
OPN	F:5'- TGAGTCTGGAAATAACTAATGTGTTTGA R:3'-GAACATAGACATAACCCTGAAGCTTTT
HMGB1 HMGB1_mut HMGB1-3’UTR	F:5'-CACTGGGCGACTCTGTGCCTCG R:3'-CGGGCCTTGTCCGCTTTTGCCA F:5'-AGACCTGACTTACATCCCCAAAAGCGTGA GCT R:3'-TTTGGGGATGTAAGTCAGGTCTTCTTTAA TGT F:5'-GCGGCTCGAGATCAATCTACTCAAAGCAT R:3'-AATGCGGCCGCAACTCCTAAGCAGATA AAC

RNA (0.2-0.5 μg) was used as template for reverse transcription using the PrimeScript reverse transcription reagent Kit (DRR037S; TaKaRa, Dalian, China). The change in RNA expression levels was determined using SYBR®Premix Ex Taq™ (DRR42OA, TaKaRa) with the ABI 9700 software (Applied Biosystems, Waltham, MA, USA) and the following conditions: denaturation at 95 °C for 10 min and 45 amplification cycles (denaturation at 95°C for 15s, annealing and extension at 60 °C for 1 min). Data analysis was performed using the comparative Ct method. Absolute quantitation of miRNA concentration in plasma samples was also directly determined using the same SYBR^®^Premix Ex Taq™ miRNA assays. The target gene mRNA results were normalized to GAPDH mRNA. For the measurement of miRNA expression, U6 or cel-miR-39 was used as an internal control (for HAVSMCs) or external control (for plasma) to normalize miRNA expression in different groups. All miRNA primers, including cel-miR-39, were purchased from RiboBio and other target genes primers were purchased from Sangon Biotech (Shanghai, China). The primer sequences are presented in Table 2 and Table 3.

#### Luciferase activity assay

The 3′-UTR of the HMGB1 mRNA fragment containing the putative miR-181b-5p-binding sequence was amplified by PCR. The primers for PCR amplification are presented in Table 2. The PCR product was inserted at the *Not* I and *Xho* I sites and the resulting construct was named WT+HMGB1-3′UTR. The HMGB1 mutant was constructed similarly to the HMGB1 wild type, except that the wild type target sequence “GAATGTA” was replaced with the mutated sequence “CTTACA”; this construct was named MUT+HMGB1-3′UTR.

The products were cloned into the pmiR-REPORTTM luciferase reporter vector and then co-transfected with miR-181b-5p mimic (50 nM) or NC mimic (50 nM). This mixture was transfected into 293T cells according to the instructions provided by the Attractene Transfection Reagent kit (Invitrogen, USA). Following transfection for 24h, the Light Switch Luciferase Assay Reagent (Dual-Glo®Luciferase Assay System, Promega, USA) was added to measure luciferase activity with an illuminometer. These experiments were performed in triplicate.

#### Clinical Study

The clinical study protocol was approved by the Hospital Ethics Committee and conformed to the principles outlined in the Declaration of Helsinki; written informed consent was obtained from all subjects.

**Table 3:** The miRNA sequences and miRBase accession numbers.

miRNA	Sequence	Number
hsa-miR-103a-3p	AGCAGCAUUGUACAGGGCUAUGA	MIMAT0000101
hsa-miR-107	AGCAGCAUUGUACAGGGCUAUCA	MIMAT0000104
hsa-miR-181b-5p	AACAUUCAUUGCUGUCGGUGGGU	MIMAT0000257

#### Subjects and protocol

A total of 182 subjects (90 men and 92 women) aged 18 to 78 years were recruited for this study from June 2016 to June 2017 at the Department of Cardiology and the Physical Examination Center of Xiangya Hospital, Central South University, Changsha, China. Patients with concomitant valvular heart disease, congenital heart disease, cardiomyopathy, acute and chronic viral or bacterial infection, asthma, tumors, and connective tissue diseases were excluded. Patients were also excluded at screening if they had a history of severe hypertension (≥180/110 mmHg) and any secondary hypertension. Additional exclusion criteria included a history of diabetes mellitus; history of cardiovascular disease, including coronary artery disease, myocardial infarction, stroke, transient ischemic attack, any revascularization procedure, congestive heart failure, or thermodynamically significant carotid or peripheral arterial disease; systemic hypertension, hypercholesterolemia; sepsis; abnormal liver and renal function or syndrome; electrolyte disturbance; and chromosomal disorders. Patients using any medications likely to affect blood pressure, including non-steroidal anti-inflammatory drugs; glucocorticoids, potent CYP3A4 inhibitors, or potassium supplements were also excluded from the study [30]. Female patients were required to not have been pregnant for 1 year. Detailed health history and demographic data including age, gender, blood pressure, fasting bloodglucose (FBG), triglyceride (TG), total-cholesterol (TC), high-density-lipoprotein-cholesterol (HDL-C), low-density-lipoprotein-cholesterol(LDL-C), alanine transaminase (ALT), aspartate transaminase (AST), blood urea nitrogen (BUN), uric acid (UA), creatinine (Cr), electro-cardiography, echocardiography, and carotid artery ultrasound were collected. Biochemical assessments were performed under fasting conditions in the early morning.

A total of 58 age-matched healthy subjects were recruited as controls. Eligible patients were 18 to 78 years old with stage 1 to 2 hypertension, either untreated or treated with an angiotensin receptor blocker (ARB). The patients without any treatment were defined as the hypertension group (n=53) and the patients treated solely with ARB were defined as the ARB group (n=71). Essential hypertension was diagnosed according to the American Joint National Committee 2014 Evidence-Based Guideline for the Management of High Blood Pressure in Adults.

#### Carotid Ultrasound Studies

Carotid ultrasound examination was performed using a USA HP SONOS5500 Color Doppler Ultrasonic Diagnosis Apparatus equipped with a 7.5-MHz transducer frequency. The electrocardiogram R wave vertex measured the posterior wall of the carotid artery lumen-intima interface (Lumen-Intima, LI) and the media membrane-adventitia membrane interface (Media-Adventitia, MA). The maximum range of LI and MA constitutes the carotid intima media thickness (IMT). Continuous measurement was performed for three cardiac cycles and the mean value served as the IMT value, which was expressed in mm.

#### Blood sampling

Peripheral blood (10 ml) was obtained from the elbow vein of all subjects and collected in tubes with EDTA. The plasma was harvested from the upper layer following centrifugation at 3000 rpm for 10min and stored at -80 °C for subsequent measurements including HMGB1, Ang II, and miRNAs.

#### Measurements of the HMGB1 and Ang II levels

The levels of HMGB1 or Ang II in culture medium or plasma were measured using a commercially available ELISA Kit (Uscn Life Science, Wuhan, China). Following the manufacturer's instructions, the absorbance was measured at 450 nm and the levels of HMGB1 or Ang II were calculated according to a standard curve.

#### Statistical analysis

All data were expressed as mean ± SEM. Patient characteristics were compared using the Student’s t test or the chi-square test. Correlations were tested by calculating the Pearson’s correlation coefficient. Comparisons of multiple groups were performed with one-way analysis of variance (ANOVA). Differences were considered statistically significant at *P* <0.05.

## RESULTS

### Up-regulation of HMGB1 in Ang II-induced phenotypic transformation of HAVSMCs

**Figure 1.**
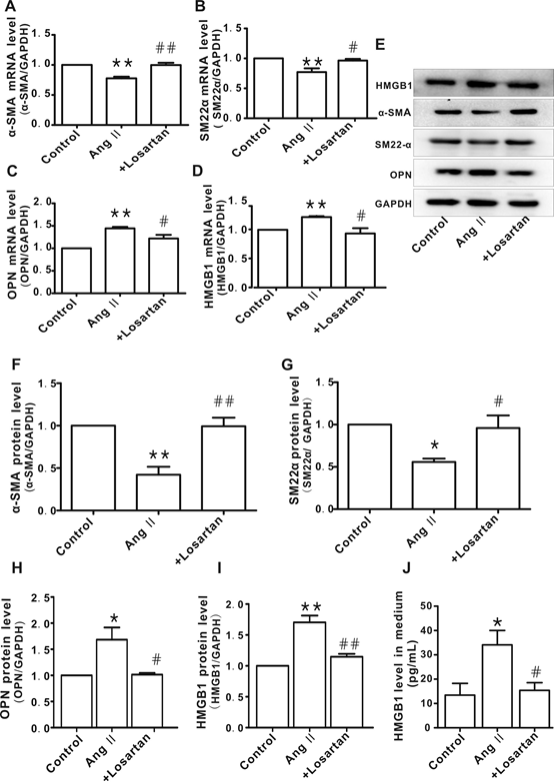
Up-regulation of high-mobility group box 1 (HMGB1) in angiotensin (Ang) II-induced human aortic vascular smooth muscle cell (HAVSMC) phenotype transformation **A-D**) mRNA levels of α-smooth muscle actin (α-SMA), smooth muscle 22α (SM22α), osteopontin (OPN), and HMGB1 in HAVSMCs, respectively. **E**) representative images of western blot analysis for HMGB1, α-SMA, SM22α, OPN, and GAPDH. **F-I**) Protein levels of α-SMA, SM22α, OPN, and HMGB1, respectively. Arbitrary optical density units of the target proteins were normalized to GAPDH and expressed as fold change. J. Protein levels of HMGB1 in culture medium *P <0.05, **P <0.01 vs. control group; ^#^*P* <0.05, ^##^*P* <0.01 vs. Ang II group. At least three independent experiments were performed for each group.

To explore the role of HMGB1 in hypertension, Ang II was used to induce the phenotypic transformation of HAVSMCs. Following incubation with Ang II (1μM) for 48h, HAVSMCs exhibited phenotypic transformation from a contractile to a synthetic type, as evidenced by down-regulation of contractile proteins α-smooth muscle actin (α-SMA) and smooth muscle 22α (SM22α) (Fig. 1 A, B, E, F, G) and up-regulation of the synthetic protein osteopontin (OPN) (Fig. 1C, E, H); these phenomena were abolished in the presence of losartan (an AT1 receptor blocker), suggesting that Ang II successfully induced the phenotypic transformation of HAVSMCs. Compared to the control group, HMGB1 mRNA and protein expression was up-regulated in Ang II-treated HAVSMCs, which was suppressed in the presence of losartan (Fig. 1D, E, I, J).

### Knockdown of HMGB1 suppressed Ang II-induced HAVSMC phenotype transformation

**Figure 2.**
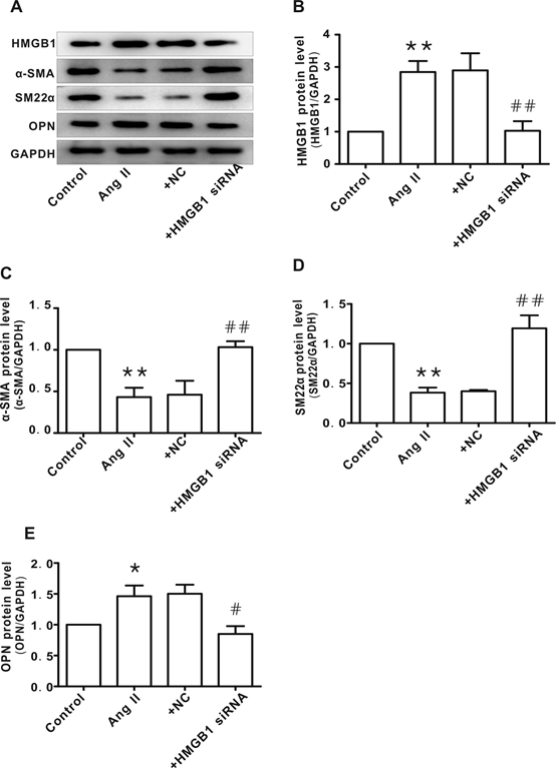
HMGB1 silencing inhibited Ang II-induced HAVSMC phenotype transformation **A**) Representative images of western blot analysis for HMGB1, α-SMA, SM22α, OPN, and GAPDH. **B-E**) Protein levels of HMGB1, α-SMA, SM22α, and OPN, respectively. Arbitrary optical density units of the target proteins were normalized to GAPDH and expressed as fold change. *P <0.05, **P <0.01 vs. control group; ^#^*P* <0.05, ^##^*P* <0.01 vs. the Ang II group. At least three independent experiments were performed for each group.

To confirm the relationship between HMGB1 and Ang II-induced HAVSMC phenotype transformation, siRNA was used to knockdown HMGB1 (Fig. 2A and B). Similar to the results with losartan, knockdown of HMGB1 attenuated Ang II-induced phenotypic transformation of HAVSMCs, as shown by the recovery of α-SMA and SM22α levels (Fig. 2C and D) as well as OPN levels (Fig. 2E). The negative control (NC) HMGB1 siRNA did not demonstrate similar effects.

### Knockdown of HMGB1 blocked Ang II-induced HAVSMC migration and proliferation

**Figure 3.**
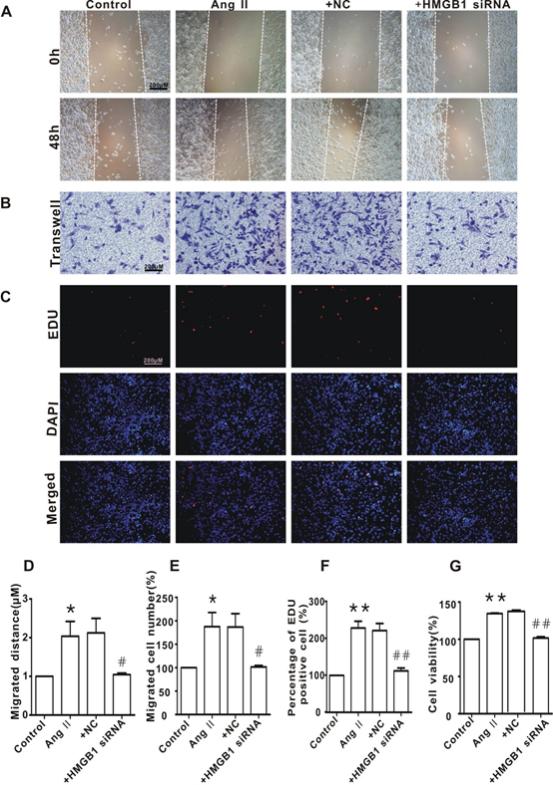
HMGB1 silencing inhibited HAVSMC migration, proliferation, and cell viability **A**) Representative images of scratch-wound healing assay (100×). **B**) Representative images of Transwell assay (100×). **C**) Representative images of Edu assay (100×). **D**) HAVSMC migration distance in each group. **E**. The number of migrated HAVSMCs in each group. **F**) The percentage of Edu-positive cells in each group. **G**. Cell viability in each group. *P <0.05, **P <0.01 vs. the control group; ^#^*P* <0.05, ^##^*P* <0.01 vs. the Ang II group. At least three independent experiments were performed for each group.

To verify the function of HMGB1 in the mediation of Ang II-induced HAVSMC phenotype transformation, cellular migration and proliferation were measured. As shown in Fig. 3A-F, silencing of HMGB1 blocked Ang II-induced HAVSMC migration, as determined by the scratch-wound healing assay (Fig. 3A and D) and Transwell chamber assay (Fig. 3B and E). Silencing of HMGB1 also inhibited HAVSMC proliferation and cells viability, as revealed by the Edu (Fig. 3C and F) and CCK8 assay (Fig. 3G). The NC HMGB1 siRNA did not display such effects.

### Identification of miR-181b-5p as an upstream regulator of HMGB1 in Ang II-treated HAVSMCs

**Figure 4.**
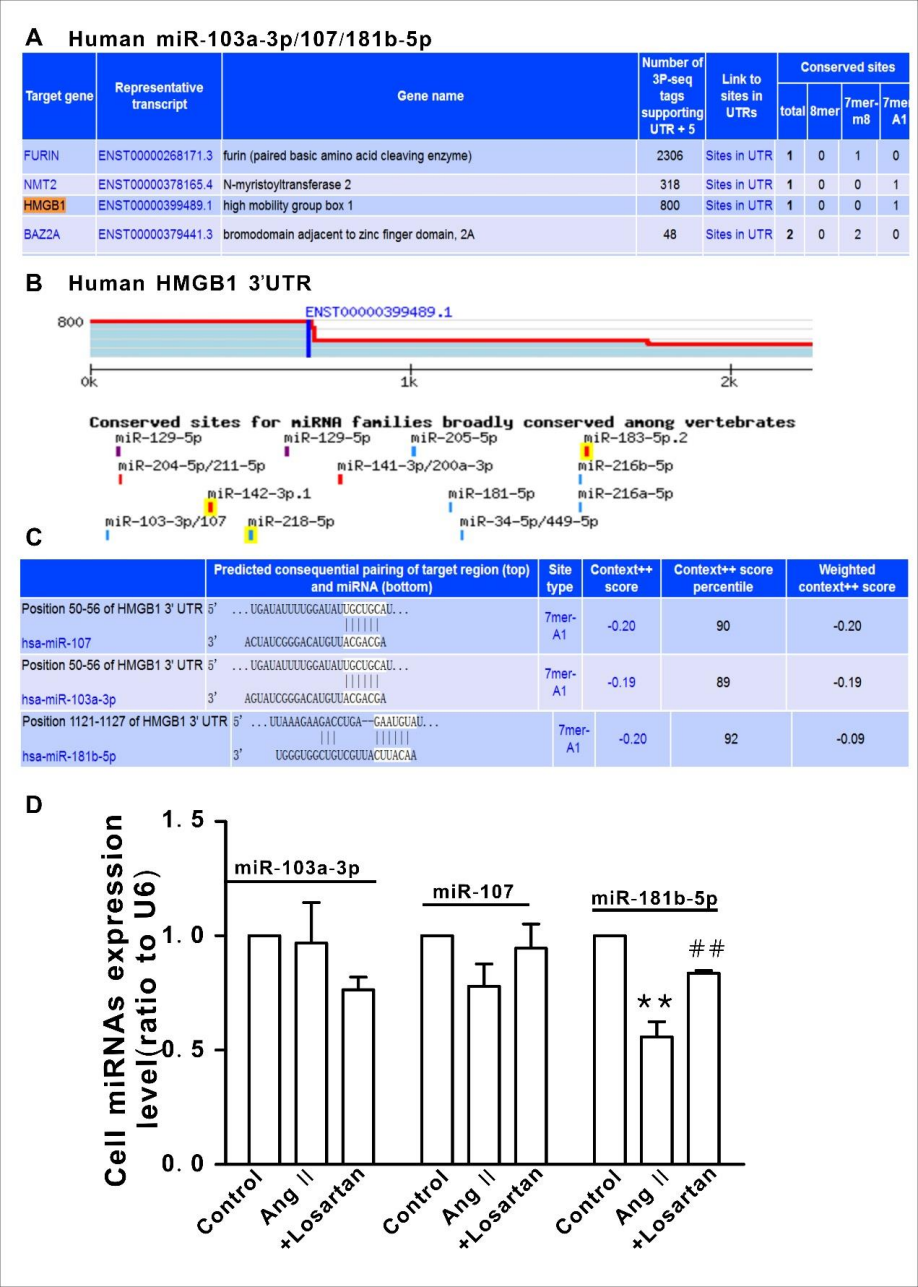
Putative binding sites of miR-181b-5p in the HMGB1 3′-UTR **A** and **B**) The three putative miRNA binding sites in the HMGB1 3′-UTR predicted by target scan analysis. **C**) Conserved putative target sites of HMGB1 in humans. **D**) Expression of the putative miRNAs in HAVSMCs. **P <0.01 vs. the control group; ^##^*P* <0.01 vs. the Ang II group. At least three independent experiments were performed for each group.

As shown in Fig. 4A and 4B, there are three putative miRNA binding sites in the HMGB1 3′-UTR (as determined by computational analysis using target scan software), which are highly conserved among humans (Fig. 4C). However, only miR-181b-5p was significantly decreased in Ang II-treated HAVSMCs and this decrease was attenuated in the presence of losartan (Fig. 4D, *P* <0.01).

**Figure 5.**
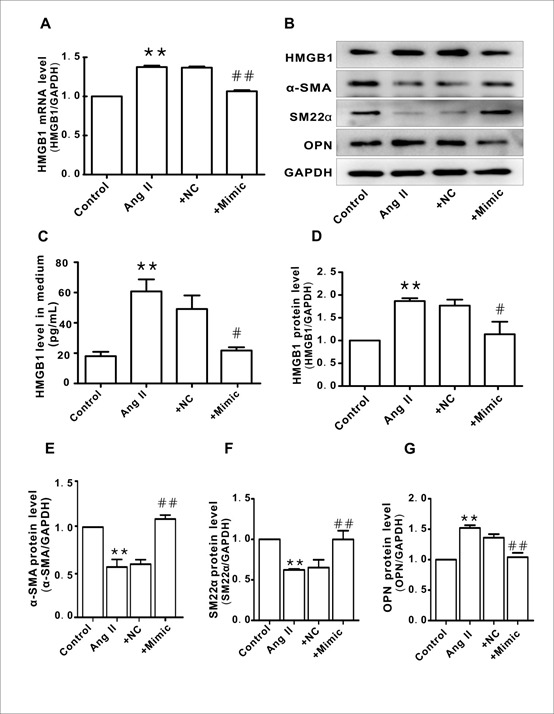
Overexpression miR-181b-5p reversed Ang II-induced HAVSMC phenotype transformation **A**. HMGB1 mRNA expression in each group. **B**. Representative images of western blot analysis for HMGB1, α-SMA, SM22α, OPN, and GAPDH. **C**. Protein levels of HMGB1 in culture medium. **D-G**. Protein levels of HMGB1, α-SMA, SM22α, and OPN, respectively. Arbitrary optical density units of the target proteins were normalized to GAPDH and expressed as fold change. *P <0.05, **P <0.01 vs. the control group; ^#^*P* <0.05, ^##^*P* <0.01 vs. the Ang II group. At least three independent experiments were performed for each group.

**Figure 6.**
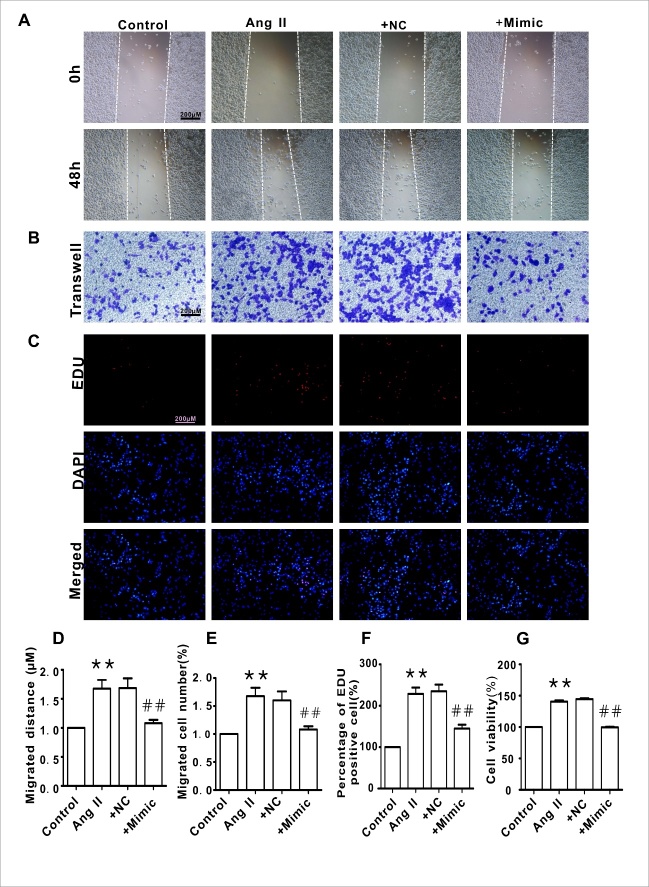
Overexpression miR-181b-5p inhibited HAVSMC migration, proliferation, and cell viability **A**) Representative images of scratch-wound healing assay (100×). **B**) Representative images of Transwell assay (100×). **C**. Representative images of Edu assay (100×). **D**) HAVSMC migration distance in each group. **E**) The number of migrated HAVSMCs in each group. **F**) The percentage of Edu-positive cells in each group. **G**) Cell viability in each group. *P <0.05, **P <0.01 vs. the control group; ^#^*P* <0.05, ^##^*P* <0.01 vs. the Ang II group. At least three independent experiments were performed for each group.

To confirm the link between miR-181b-5p and HMGB1, miR mimics were applied. As shown in Fig. 5, the miR-181b-5p mimic markedly suppressed HMGB1 expression (Fig. 5A-D) and reversed the phenotype transformation in Ang II-treated HAVSMCs, as evidenced by the recovery of α-SMA and SM22α levels (Fig. 5E and F) as well as OPN levels (Fig. 5G). Similarly, the miR-181b-5p mimic also blocked Ang II-induced HAVSMC migration (Fig. 6A, B, D, E) and proliferation (Fig. 6C, F, G). The NC of the miR-181b-5p mimic did not exert any obvious effect on HMGB1 expression and phenotypic transformation.

**Figure 7.**
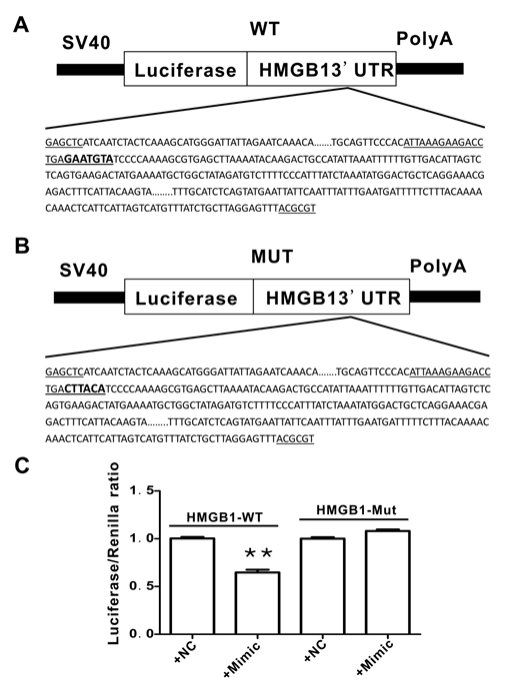
Transcriptional effect of the miR-181b-5p mimic on the 3′-UTR of HMGB1 in HAVSMCs **A**) The 3′-UTR of wild type (WT) HMGB1 (the potential binding site for miR-181-5p is indicated in bold letters). **B**) The 3′-UTR of the mutated (MUT) HMGB1 (the MUT miR-181-5p binding site is indicated in bold letters). **C**) Luciferase activity in HAVSMCs in the presence of the miR-181b-5p mimic. At least three independent experiments were performed for each group.

To confirm that HMGB1 is a direct target of miR-181b-5p, dual luciferase reporter vectors containing wild-type (WT) or mutant HMGB1 3'-UTR were constructed (Fig. 7A and B). The results of the luciferase assay indicated that miR-181b-5p inhibited luciferase activity in the HMGB1 WT 3'-UTR group; however, the inhibitory effects were abolished when the HMGB1 3'-UTR binding site was mutated (Fig. 7C).

### Clinical characteristics

This study specifically focused on essential hypertension. The clinical study included 182 subjects in total; these were divided into three groups based on clinical circumstances and research objectives as follows: healthy controls served as the normal group (n=58), patients not receiving any anti-hypertension medication served as the hypertension group (n=53), and patients treated solely with an angiotensin receptor blocker (ARB) served as the ARB group (n=71).

**Figure 8.**
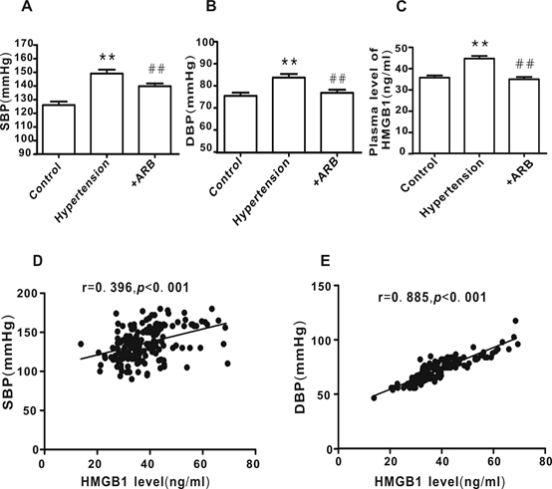
Correlation between HMGB1plasma levels and blood pressure **A** and **B**) Subject blood pressure. **C** HMGB1plasma levels. **D** and **E**) Correlation between HMGB1 plasma levels of and blood pressure. Control group, n=58; hypertension group, n=51; +ARB treatment group, n=71. **P <0.01 vs. control, ^##^*P* <0.01 vs. hypertension.

There were no significant differences in gender, age, FBG, TG, TC, HDL-C, LDL-C, ALT, AST, BUN, UA and Cr between the three groups, except for the systolic blood pressure (SBP) and diastolic blood pressure (DBP). Compared to the control group, the SBP and DBP were obviously elevated in the hypertensive patients; these values were significantly decreased subsequent to ARB (such as losartan, valsartan, or Irbesartan) administration for over three months (Table 4, Fig. 8A and B).

### Positive correlation between the plasma level of HMGB1 and blood pressure

HMGB1 plasma levels were significantly elevated in hypertensive patients compared with the healthy controls; this was dramatically reversed following treatment with ARB (Fig. 8C; *P* <0.01). As shown in Fig. 8D and E, there was a significant positive correlation between HMGB1 plasma levels and SBP (r= 0.396, *P* <0.001) or DBP (r=0.885, *P* <0.001).

### Clinical relevance of the miR-181b-5p/HMGB1 pathway in hypertension

**Figure 9.**
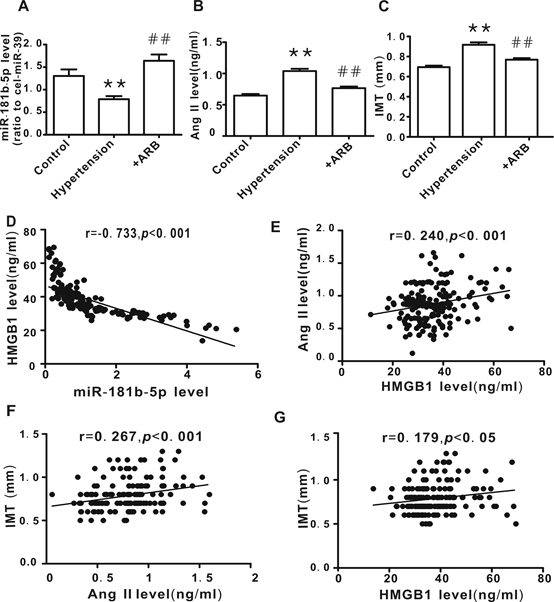
Correlation between miR-181b-5p, HMGB1, Ang II, and intima-media thickness (IMT) in hypertensive patients **A**) miR-181b-5p plasma levels. **B**) Ang II plasma levels. **C**. IMT values, determined by carotid artery ultrasound. **D**) The correlation between HMGB1 level and miR-181b-5p level. E) The correlation between HMGB1 level and Ang II level. **E**) The correlation between IMT and Ang II level. **F**) The correlation between IMT and HMGB1 level. Control group, n=58; Hypertension group, n=51, Hypertension +ARB group, n=71. **P <0.01 vs. control, ^##^*P* <0.01 vs. Hypertension.

To evaluate the clinical relevance of the miR-181b-5p/HMGB1 pathway in hypertension, the correlations between miR-181b-5p, HMGB1, Ang II, and intima-media thickness (IMT, an index for carotid arterial remodeling) were analyzed. Consistent with the elevated HMGB1 levels in hypertension patients, the plasma level of miR-181b-5p was decreased concomitant with an increase in Ang II levels and IMT.These phenomena were attenuated in hypertensive patients following ARB treatment (Fig. 9A-C). There was a negative correlation between miR-181b-5p and HMGB1 (Fig. 9D), while a positive correlation was observed between Ang II and HMGB1 (Fig. 9E), IMT and HMGB1 (Fig. 9G), and IMT and Ang II (Fig. 9F).

## DISCUSSION

In the present study, using the in vitro Ang II-induced phenotypic transformation model of vascular smooth muscle cells, we demonstrated that HMGB1 was up-regulated, accompanied by down-regulation of contractile proteins and up-regulation of synthetic proteins. Both losartan and HMGB1 siRNA were able to attenuate Ang II-induced HAVSMC phenotypic transformation, migration, and proliferation. Bioinformatics analysis identified miR-181b-5p as an upstream regulator of Ang II-induced HMGB1 expression; miR-181b-5p mimic produced similar results to losartan and HMGB1 siRNA. The results of the dual luciferase reporter gene assay demonstrated the direct interaction between miR-181b-5p and HMGB1. The findings of the clinical study showed that the plasma level of miR-181b-5p was decreased, while HMGB1 and Ang II levels were increased in hypertensive patient’s concomitant with an increase in IMT, a vascular remodeling index. These phenomena were attenuated in patients who received angiotensin receptor blockers. There was a negative correlation between the plasma levels of miR-181b-5p and HMGB1. To the best of our knowledge, this is the first study to provide evidence that the miR-181b-5p/HMGB1 pathway may contribute to the phenotypic transformation of vascular smooth muscle cells and vascular remodeling in hypertensive patients.

**Table 4:** Baseline characteristics of participants.

	Control (n=58)	Hypertension (n=53)	+ARB (n=71)
Age, yrs	59.28±1.47	60.83±1.43	61.48±1.32
Male (% of total)	29(15.9%)	27(14.8%)	34(18.7%)
Female (% of total)	29(15.9%)	26(14.3%)	37(20.7%)
SBP(mmHg)	124.36±2.48	146.42±2.78**	137.35±1.98##
DBP(mmHg)	74.24±1.35	82.04±1.51**	75.54±1.32##
FBG, mmol/L	5.49±0.07	5.53±0.08	5.50±0.08
TG, mmol/L	1.46±0.08	1.50±0.80	1.56±0.08
TC, mmol/L	5.14±0.11	5.31±0.10	5.33±0.08
HDL, mmol/L	1.38±0.04	1.27±0.05	1.35±0.05
LDL, mmol/L	3.09±0.12	2.64±0.11	2.84±0.29
ALT, U/L	26.02±1.55	26.35±2.48	26.99±2.38
AST, U/L	22.50±1.02	22.99±1.11	23.60±1.17
BUN, mmol/L	5.24±0.17	5.29±0.21	5.62±0.14
UA, mmol/L	348.97±11.53	349.92±12.90	355.41±11.26
Cr, μmol/L	84.07±1.22	85.19±1.07	85.75±0.81

Data presented as mean ± SEM.

** *P* lt;0.01 vs. control,

## *P* <0.01 vs. hypertension. Abbreviations: Control for the healthy control people (n=58); hypertension for the essential hypertensive patients without any drugs(n=53); +ARB, the essential hypertensive patients only with angiotensin II receptor blockage for treatment(n=71); SBP, systolic blood pressure; DBP, diastolic blood pressure; FBG, fasting blood glucose; TG, Triglyceride; TC, total cholesterol; HDL-C, high density lipoprotein cholesterol; LDL-C, low density lipoprotein cholesterol; ALT, Alanine transaminase; AST, Aspartate transaminase; BUN, blood urea nitrogen; UA, uric acid; Cr, creatinine.

Vascular remodeling in hypertension may initially be adaptive; however, eventually it becomes maladaptive and contributes to the development and complications of hypertension[28, 30].VSMCs phenotypic transformation is a major initiating factor of vascular remodeling in hypertension[10]. Under pathological conditions, VSMCs accelerate the phenotypic transformation from contractile to synthetic phenotype. This phenotypic switching obviously changes the functions of VSMCs[31]. For example, synthetic VSMCs migrate and proliferate more easily than contractile VSMCs. Furthermore, synthetic VSMCs can synthesize up 25 to 46-fold more collagen than contractile VSMCs [11, 32]; all of which are closely related to vascular remodeling in hypertension.

It is well known that the renin-angiotensin system is activated in most cases of essential hypertension and plays a key role in blood pressure elevation[33]. In essential hypertension, Ang II plasma levels are usually elevated. Thus, ACEI and angiotensin receptor blockers constitute first-line treatments of anti-hypertension. There is evidence that Ang II is able to induce VSMCs phenotypic transformation[34, 35], which increases the proliferation and migration ability of VSMCs, resulting in vascular remodeling[32, 36]. Consistent with these reports, the results of the present study demonstrated a down-regulation of contractile proteins and an up-regulation of synthetic proteins in Ang II-treated HAVSMCs, concomitant with an increase in cellular migration and proliferation. These phenomena were attenuated in the presence of losartan, indicating that the in vitro model of Ang II-induced VSMCs phenotypic transformation was successful.

Hypertension has been described as an inflammatory disease and vascular inflammation is considered to play a critical role in vascular remodeling in several vascular diseases such as hypertension and atherosclerosis[37]. Both in vivo and in vitro studies have demonstrated that HMGB1 is critical for the development of vascular inflammation [36, 38, 39]. In addition, Ang II has also been shown to be involved in vascular inflammation [31, 40, 41]. However, whether there is a correlation between Ang II and HMGB1 unkown. In the present study, we found that HMGB1 expression (mRNA and protein) was significantly up-regulated in Ang II-treated HAVSMCs and that this was reversed in the presence of losartan, confirming a link between Ang II and HMGB1. As HMGB1 plays an important role in many pathogenic processes, including VSMCs abnormal proliferation and migration[42], we hypothesize that HMGB1 has a key role in mediating Ang II-induced VSMCs phenotypic transformation, cellular migration, and proliferation. The results of this study demonstrate that knockdown of HMGB1 dramatically attenuated Ang II-induced phenotypic transformation of HAVSMCs, cellular migration, and proliferation, supporting our hypothesis.

To elucidate the mechanisms responsible for the up-regulation of HMGB1 in Ang II-treated HAVSMCs, we used bioinformatics software to predict potential miRNAs targeting HMGB1; three candidate miRNAs (miR-103a-3p, miR-107, and miR-181b-5p) were identified. In Ang II-treated HAVSMCs, only miR-181b-5p was dramatically down-regulated; this effect was reversed in the presence of losartan, suggesting that miR-181b-5p might be an upstream negative regulator of HMGB1. Using miR-181b-5p mimic, we found that HMGB1 mRNA and protein expression was suppressed in Ang II-treated HAVSMCs, confirming the role of miR-181b-5p in the suppression of HMGB1expression. The results of the dual luciferase reporter gene assay indicated a direct interaction between miR-181b-5p and HMGB1.

To extend the clinical relevance of our cell study-based findings, we carried out a clinical study. Our results showed that the plasma levels were significantly elevated in hypertensive patient’s concomitant with an increased vascular remodeling index(IMT), demonstrating a positive correlation between HMGB1 levels and IMT or blood pressure. These results suggest that HMGB1 may also contribute to vascular remodeling in essential hypertension. Interestingly, a number of studies have reported that plasma levels of HMGB1 were elevated in pulmonary arterial hypertension (PAH) and contributed to the vascular remodeling of PAH [40, 43, 44]. Our current results indicate that HMGB1 has the same function in essential hypertension as in PAH. Our cell study demonstrated that HMGB1 mediated Ang II-induced phenotypic transformation of HAVSMCs. Moreover, we found a positive correlation between the plasma levels of HMGB1 and Ang II. Following treatment with an angiotensin receptor blocker, the plasma levels of HMGB1 in hypertensive patients were obviously reduced, confirming a link between Ang II and HMGB1. Finally, we examined the plasma levels of miR-181b-5p in hypertensive patients. The results showed that miR-181b-5p levels were markedly decreased and inversely correlated with the plasma levels of HMGB1. This decrease in miR-181b-5p levels accompanied by an increase in HMGB1 was reversed in hypertensive patients treated with angiotensin receptor blockers, further verifying the function of miR-181b-5p as a negative regulator of HMGB1. Of note, although the clinical study limited us to examining the expression of miR-181b-5p, HMGB1, α-SMA, SM22α, and osteopontin in the blood vessels, it still supports our in vitro study findings.

In summary, the results presented here demonstrate for the first time that HMGB1 is up-regulated in essential hypertension, which contributes to vascular remodeling by modulating the phenotypic transformation of smooth muscle cells from contractile to synthetic type under the activation of the renin-angiotensin system. We have also identified miR-181b-5p as a negative regulator of HMGB1 expression. Thus, Ang II/ miR-181b-5p/ HMGB1 might form a novel pathway involved in the regulation of vascular remodeling in hypertension. Interfering with this pathway may have therapeutic and preventive value for hypertension.

### Limitation of the study

This study had a major limitation that needs to be acknowledged and addressed. Although the miR-181b-5p/HMGB1 pathway was shown to be involved in Ang II-induced phenotypic transformation of smooth muscle cell in vitro, the clinical study limited verification of the involvement of the miR-181b-5p/HMGB1 pathway in phenotypic transformation of smooth muscle cells to the blood vessels of hypertensive patients. These issues will be solved using proper hypertension animal models in our future studies.
